# Pre-tibial myopericytoma: a case report

**DOI:** 10.1093/jscr/rjac021

**Published:** 2022-02-02

**Authors:** Claudia J K Cockburn, Elliot J D Crene, William J Cockburn

**Affiliations:** 1 Intensive Care Unit, GCUH, Gold Coast, Australia; 2 Neurosurgery, GCUH, Gold Coast, Australia; 3 Plastic Surgery, Brisbane, RBWH, Brisbane, Australia

## Abstract

Myopericytoma (MPC) is a rare, benign tumour often presenting as a cutaneous growth commonly in the lower extremities. It is distinguished by its concentric layering of spindle shaped myoid appearing cells perivascularly. These cells diagnostically stain positive to alpha smooth-muscle actin and rarely positive to desmin stain. This case study reviews the presentation of a 56-year-old male with a slow-growing, pre-tibial lesion developing over a 7–8 year period. This lesion was asymptomatic and demonstrated vascular involvement on ultrasound scan. This lesion measured 19 × 15 × 9 mm histologically and contained bland spindle cells surrounding vessels that interestingly stained positive to both alpha smooth-muscle actin and desmin. The histological findings in correlation to clinical presentation and imaging led to a diagnosis of MPC.

## INTRODUCTION

Myopericytoma (MPC) is a rare, benign, slow-growing tumour that most commonly presents on the lower extremities [1]. These lesions usually contain thin-walled vessels with surrounding plump spindle to round myxoid cells arranged in a concentric pattern [2]. Blood vessels often demonstrate a characteristic ‘stag-horn’ appearance. Microscopic analysis of MPCs often demonstrates well-circumscribed lesions with occasional prominent atypia and mitotic activity [3]. MPCs are commonly painless lesions that affect the skin, usually the dermis and subcutaneous tissue [4]. Diagnosis is made through laboratory staining of tissue samples with MPCs being positive for alpha smooth-muscle actin, h-caldesmon and rarely focal desmin. The tumour described in this study interestingly tested positive for desmin staining.

## CASE REPORT

A 56-year-old male presented with a 7–8 year history of a slow-growing firm lump on his right distal tibia, midline on his anterior leg. The patient was otherwise in good health with no history of previous skin lesions and no family history of same. The lesion was subcutaneous and largely painless with no cutaneous manifestations. On physical examination, the nodule was 27 × 22 mm pre-tibial, 6 cm from the ankle joint. It was firm to palpation, subcutaneous with no skin discolouration or deformation. Ultrasound scan of the right lower leg demonstrated a well-defined, oval hypoechoic solid lesion with internal vascularity in the subcutaneous tissues measuring 18 × 7 × 20 mm. Biopsy was not performed due to vascularity of the lesion. Presumed diagnosis included fibroma, schwannoma or neurofibroma. The lesion was excised with clear margins. Microscopic examination revealed a well circumscribed, unencapsulated spindle cell lesion associated with varying sized dilated congested vessels, some of which showed a haemangiopericytomatous (HMP) pattern. The stroma included bland spindle cells seen surrounding blood vessels. No evidence of necrosis, atypia or mitotic activity was visualised. Further investigation with immunohistochemistry demonstrated spindle cells positive for both alpha smooth-muscle actin and desmin, with desmin showing variable positivity. The tumour cells were negative for CD-34. The examination, investigation and pathology are in keeping with a diagnosis of MPC. The lesion as stated above was completely excised, the wound demonstrated good recovery and no further surveillance required.

## DISCUSSION

MPC is a rare, cutaneous, benign lesion that has only recently been described in the medical literature. Its microscopically distinctive features include its perivascular concentric layering of stromal cells [5]. MPCs comprise a mixture of solid cellular areas comprised of oval and spindle shaped cells and vascular channels with prominent branches providing the tumour with both a myofibroma and haemangiopericytoma-like appearances retrospectively [6]. Depending on the amount of solid to vascular area, can provide great range in histological appearance of MPCs from myofibromatosis to angioleiomyoma and glomus tumours [5, 7]. Glomus tumours do demonstrate perivascular arrangement of cells however are not concentrically arranged [4]. Angioleiomyomas demonstrate abundant vascular channels but a differentiated from MPC as they are usually painful lesions and histologically present smooth-muscle fascicles, which will stain positive for desmin [8]. MPC tumours are rarely infiltrative and recurrence is rare [6]. It has been suggested that the malignant potential of MPC is strongly associated with the depth of the tumour however only a limited number of cases have been reported and more need to be reviewed with a more prolonged follow up to substantiate this hypothesis [4, 7].

MPCs tumours were first described in a case study by Dictor *et al.* in 1992 who noted myofibromatosis-like haemangiopericytomas in the thyroid of a 5-year-old boy [9]. In this article, Dictor *et al.* proposed these cells are termed ‘myoperiocytes’ following review with electron microscope and immunohistochemical analysis [9]. Later, in 1998, Granter *et al.* further described myoperiocytoma after examining 24 subcutaneous tumours [9]. Granter *et al.* describe the distinguishing feature from myofibroma being the distinctive periluminal proliferation of round to oval cells lying in a concentric pattern [10].

MPCs most commonly present as slow-growing painless firm lesions that are well circumscribed [7]. Mentzel *et al.* reviewed 54 cases of MPCs and identified these lesions to most frequently present in the skin and soft tissues of lower extremities and occur more frequently in males than females in a wide age bracket [6]. This is in keeping with this case review being a middle-aged male presenting with a pre-tibial painless growth, slow-growing over a 7–8 year period. Interestingly, Mentzel *et al.* identified only three cases positive to desmin stain [6]. All cases studied demonstrated positive stain to ASMA. This case study presents a rare form of MPC, which stains positive to desmin. Desmin is a stain used to confirm myogenic origin of tumours and helps differentiate tumours from myofibroma and myxoma, which do not stain desmin positive [11]. As stated previously, angioleiomyomas often stain positive for desmin while <9% of MPC cases stain positive. This suggests that MPC is composed of more immature cells than angioleiomyomas. Whether a positive desmin stain of MPC has any correlation with the maturity of the cells remains to be determined.

This case demonstrates a classical presentation of the rare tumour, MPC, which was completely excised surgically with good wound recovery. Interestingly, this was a rare form of the tumour demonstrating uptake of desmin on immunohistochemistry staining in the pathology laboratory.
